# Modulation of TRP channels by resveratrol and other stilbenoids

**DOI:** 10.1186/1744-8069-9-3

**Published:** 2013-02-15

**Authors:** Lina Yu, Shenglan Wang, Yoko Kogure, Satoshi Yamamoto, Koichi Noguchi, Yi Dai

**Affiliations:** 1 Department of Pharmacy, School of Pharmacy, Hyogo University of Health Sciences, 1-3-6 Minatojima, Chuo-ku, Kobe, Hyogo 650-8530, Japan; 2 Department of Anatomy and Neuroscience, Hyogo College of Medicine, Nishinomiya, Hyogo 663-8501, Japan; 3 Traditional Medicine Research Center, Chinese Medicine Confucius Institute at Hyogo College of Medicine, Kobe, Hyogo 650-8530, Japan

## Abstract

**Background:**

Resveratrol (3,5,4’ - trihydroxy-trans-stilbene), a widely distributed natural stilbenoid, was proposed to account for the unique effects of red wine on life span and health. It has been reported to possess various biological and pharmacological activities, such as anti-oxidant, anti-inflammatory, and anti-carcinogenic effects. Here, using whole-cell patch-clamp techniques and behavioral analyses, we investigated whether resveratrol and other stilbenoids can modulate TRP channels in sensory neurons *in vitro,* and have analgesic effects *in vivo*.

**Results:**

We found that resveratrol dose-dependently suppressed the allyl isothiocyanate (AITC)-induced currents (*I*_AITC_) in HEK293 cells that express TRPA1, as well as in rat dorsal root ganglion (DRG) neurons. Instead, pinosylvin methyl ether (PME), another derivate of stilbene which has a similar structure to resveratrol, dose-dependently blocked the capsaicin-induced currents (*I*_CAP_) in HEK293 cells that express TRPV1 as well as in DRG neurons. Interestingly, resveratrol had no inhibitory effect on the *I*_CAP_, and PME had no effect on the *I*_AITC_. Otherwise, trans-stilbene showed no any effect on *I*_AITC_ or *I*_CAP_. The concentration response curve of AITC showed that resveratrol inhibited the action of TRPA1 not by changing the EC_50_, but by suppressing the AITC-induced maximum response. By contrast, the inhibition of TRPV1 by PME did not change the capsaicin-induced maximum response but did cause a right shift of the EC_50_. Moreover, pre-administration of resveratrol suppressed intraplantar injections of AITC-evoked nocifensive behaviors, as well as that PME suppressed capsaicin-evoked one.

**Conclusions:**

These data suggest that resveratrol and other stilbenoids may have an inhibitory effect on TRP channels. In addition, these stilbenoids modulate TRP channel activity in different ways.

## Background

**Figure 1:**
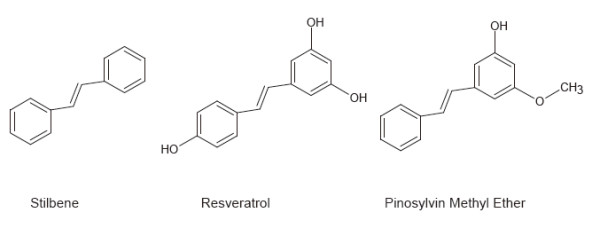
Chemical structures of trans-stilbene, resveratrol and pinosylvin methyl ether.

Stilbenoids are bioactive compounds that show beneficial effects on human health, such as anti-tumor activity and increased rate of survival. Resveratrol (3,5,4’ - trihydroxy-trans-stilbene) is a representative stilbenoid which consists of two aromatic rings that are attached by a methylene bridge (Figure 1). It is a natural phenol and phytoalexin, produced biologically by 72 different plant species, especially grapevines, pine trees, and legumes [1]. Resveratrol was first isolated from the roots of white hellebore (*Veratrum grandiflorum O. Loes*), and from the roots of *Polygonum cuspidatum*, a plant used in traditional Chinese and Japanese medicine [2,3]. The concentration of resveratrol in grapes skin is about 0.1 mg/100 g of fresh weight [4]. Resveratrol has been reported to have potent anti-aging, anti-inflammatory, anti-cancer anti-oxidative, and chemo-protective characteristics [5-11]. Recently, some studies have indicated that resveratrol has analgesic properties against both acute and chronic pain that is triggered by nocifensive stimuli, inflammation, or nerve-injury [9-14]. Mechanisms of this analgesic action have been suggested to be alterations of the expression of serum tumor necrosis factor-alpha, whole brain nitric oxide in the diabetic rat model [14], reduction of expression of cyclooxygenase-2 in the inflammatory pain model [10], or inhibition of cyclin-dependent kinase 5 activity in primary afferent neurons [15]. However, the direct molecular target of resveratrol has been elusive.

Since the discovery of the *Drosophila melanogaster* transient receptor potential (*trp*) gene, it has become known that mammalian genomes contain 28 homologous genes encoding proteins that are capable of forming a large variety of cationic channels, usually with permeability to sodium and calcium [16]. TRPV1 and TRPA1 are members of branch V and A of the TRP family of cation channels respectively, and are expressed by a subset of small-sized DRG or trigeminal ganglia neurons in neonatal rats, adult rats and mice [17,18]. It is well known that TRPV1 is activated by capsaicin, protons, or heat (with a thermal threshold > 43°C), which cause pain *in vivo*[17,19]. Analyses of mice lacking TRPV1 have shown that it is essential for selective modalities of pain sensation, as well as for tissue injury-induced thermal hyperalgesia [20]. Like TRPV1, TRPA1 is activated by various kinds of noxious compounds (such as allyl isothiocyanate (AITC), cinnamaldehyde, cannabinoids, and allicin) through their ability to covalently modify cysteine residues in the channel proteins [21-26]. TRPA1 has also been reported to be activated by bradykinin, intracellular alkalization, and the endogenous aldehyde, 4-hydroxynonenal [21,27,28]. Studies using knockout mice demonstrated that TRPA1 is an important component of the transduction machinery through which environmental irritants and endogenous proalgesic agents depolarize nociceptors to elicit inflammatory pain [29]. There is no doubt that TRPV1 and TRPA1 are critical molecules for the detection and modulation of pain sensations.

Using patch-clamp and behavior analyses, we investigated the pharmacological effects of resveratrol and related stilbenoids on these two pain-related TRP channels, TRPV1 and TRPA1. We observed that resveratrol significantly inhibited *I*_AITC_, but not *I*_CAP_, both in heterologous HEK293 cells and DRG neurons. Resveratrol also inhibited AITC-induced nocifensive behavior in adult rats. By contrast, pinosylvin methyl ether (PME), an analog of resveratrol, inhibited *I*_CAP_ and nocifensive behavior. The pathway targeted by resveratrol and other related stilbenoids may become useful for developing treatments for many painful diseases in the future.

## Results

### Resveratrol suppresses *I*_AITC_ but not *I*_CAP_ in heterologous HEK293 cells

**Figure 2:**
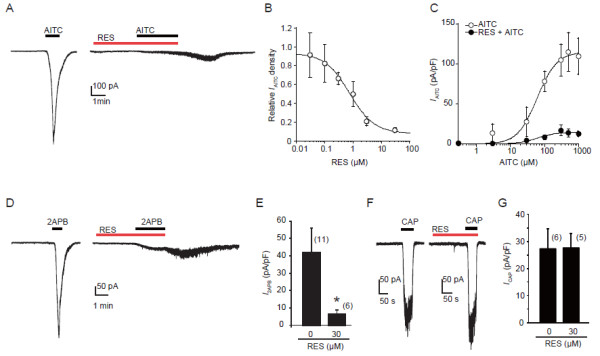
**Resveratrol suppresses TRPA1 currents, but not TRPV1 currents in a concentration dependent manner in transfected HEK293 cells. A**: Representative traces from whole-cell patch-clamp experiments show the *I*_AITC _(AITC, 100 μM) in the absence (left) or presence (right) of resveratrol (RES, 30 μM). **B**: Dose–response curve showing inhibition of *I*_AITC _by RES. Each point represents relative *I*_AITC _with different concentration of RES treatment normalized to the *I*_AITC _without RES treatment (mean ± SEM, n = 6–11). IC50 = 0.75 μM. **C**: Concentration curves of *I*_AITC _in the absence (open circles) and presence (filled circles) of RES. Note the maximum response was inhibited without changing the EC50 (61.2 μM for AITC, 61.7 μM for RES + AITC). **D**: Representative traces show the *I*_2APB _(2APB, 400 μM) in the absence (left) or presence (right) of resveratrol (RES, 30 μM). **E**: The bars graph shows RES significantly inhibits *I*_2APB _density (pA/pF). * p < 0.05, unpaired *t *– test. **F**: Representative traces show the *I*_CAP _(capsaicin, 20 nM) in the absence (left) or presence (right) of RES. **G**: Bars graph shows no effect of RES on *I*_CAP_. Numbers in parenthesis indicate cells tested. AITC or capsaicin was perfused until current reaching the peak. Holding potential (Vh) = −60 mV in all experiments.

We examined the effects of resveratrol on the *I*_AITC_ in HEK293 cells expressing mTRPA1. The AITC (100 μM) did not induce any significant current in untransfected HEK293 cells. In mTRPA1 expressing HEK293 cells, the *I*_AITC_ (AITC, 100 μM) underwent a fast activation component and then by rapid inactivation (desensitization). After 3 min pretreatment of resveratrol (30 μM), not only was the time of the activation component clearly extended, but the magnitude of *I*_AITC_ was significantly suppressed (Figure 2A). The inhibition of *I*_AITC_ by resveratrol appeared from a low concentration (0.3 μM) with an IC_50_ value of approximately 0.75 μM (Figure 2B).

To examine how resveratrol changes TRPA1 responsiveness, we measured *I*_AITC_ density by applying a range of concentrations of AITC in the absence or presence resveratrol. We found that treatment with resveratrol (30 μM, 3 min) strongly suppressed *I*_AITC_ at the maximum response (−115.2 ± 5.9 pA/pF for AITC vs −15.1 ± 1.8 pA/pF for AITC with resveratrol) without affecting EC_50_ (61.2 μM for AITC vs 61.7 μM for AITC with resveratrol) (Figure 2C). 2-aminoethoxy diphenyl borate (2APB), as a non-electrophilic agonist, is known to activate TRPA1 without covalent modification of the cysteine residues. To further investigate the inhibition mechanism, the effect of resveratrol on 2APB-induced currents in mTRPA1-transfected HEK293 cells was tested. We found that resveratrol also significantly reduced 2APB-induced current in HEK293 cells expressing mTRPA1 (−42.1 ± 14.0 pA/pF for 2APB without resveratrol, n=11; -6.4 ± 2.4 pA/pF for 2APB with resveratrol, n=6, p < 0.05) (Figure 2D, E). We confirmed that application of resveratrol alone at less than 30 μM causes no change of membrane currents in HEK293 cells expressing mTRPA1. These data clearly indicate a suppressive effect of resveratrol on TRPA1 channel activity, and this effect was not dependent on agonist’s electrophilicity. We also tested if resveratrol had any pharmacological actions on another pain-related TRP channel, TRPV1. In contrast to TRPA1, resveratrol at 30 μM did not significantly inhibit *I*_CAP_ (capsaicin, 20 nM) in HEK293 cells expressing rTRPV1 (Figure 2F, G) (−27.3 ± 7.3 pA/pF for capsaicin without resveratrol, n=6; -27.7 ± 5.4 pA/pF for capsaicin with resveratrol, n=5, *p* > 0.05).

### Resveratrol suppresses *I*_AITC_ in DRG neurons

**Figure 3:**
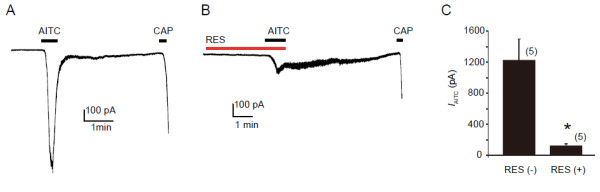
**Resveratrol suppresses *I***_**AITC **_**in DRG neurons. A**, **B**: Representative traces show *I*_AITC _in the absence (**A**) or presence (**B**) of resveratrol (RES, 30 μM). **C**: Resveratrol significantly inhibits *I*_AITC _(pA). Numbers in parenthesis indicate cells tested. * p < 0.05, unpaired *t *- test. AITC was perfused until current reaching the peak. Holding potential (Vh) = −60 mV in all experiments.

Because cell types differ in their membrane composition, we asked whether resveratrol would also suppress TRPA1 channels in sensory neurons. We then tested the effect of resveratrol on *I*_AITC_ in rat DRG neurons. AITC, as a potent activator of TRPA1, has been used in patch-clamp experiments to detect the TRPA1 current. A previous study indicated that while there are AITC-sensitive cells in TRPA1 knockout mice, these are not where the TRPA1 is expressed. To confirm the *I*_AITC_ in the DRG is a TRPA1-mediated event, capsaicin at 1 μM was applied at the end of recording using the patch-clamp preparation (see Methods section). We observed that AITC at 300 μM induced a large inward current in DRG neurons (Figure 3A). After treatment with resveratrol at 30 μM for 3 min, *I*_AITC_ were strongly suppressed (Figure 3B, C) (−1228.8 ± 270.9 pA, n=5 for AITC without resveratrol treatment, vs -127.7 ± 20.8 pA, n = 5 for AITC with resveratrol treatment, *p* < 0.05). We confirmed that application of resveratrol alone at 30 μM caused no change of membrane currents in DRG neurons. Together, these data indicate that resveratrol suppress *I*_AITC_ both in a heterologous expression system and in sensory neurons.

### PME suppresses *I*_CAP_ but not *I*_AITC_ in heterologous HEK293 cells and DRG neurons

**Figure 4:**
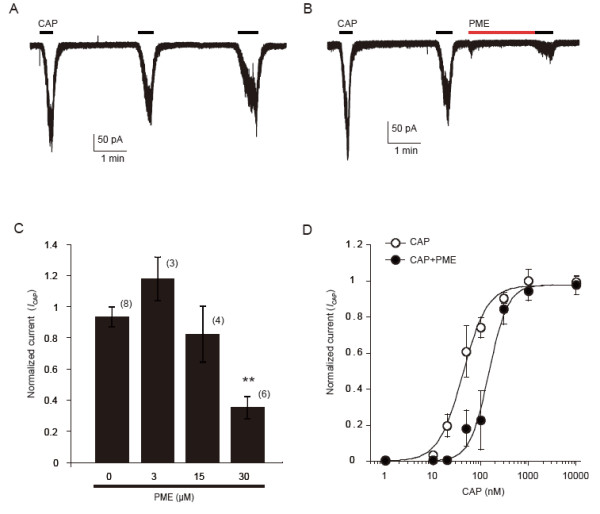
**PME suppresses *I***_**CAP **_**in transfected HEK293 cells. A**, **B**: Representative trace shows *I*_CAP _(capsaicin, 20 nM) in the absence (**A**) or presence (**B**) of PME (30 μM). Cells were perfused for 2 min with solution containing PME before reapplication of the capsaicin (CAP). **C**: PME inhibit *I*_CAP_ dose dependently. Numbers in parentheses indicate cells tested. ** p < 0.01 versus 0 μM PME, one-way ANOVA followed by Fisher’s PLSD. **D**: *I*_CAP _concentration-response curves for TRPV1 activation in the absence (open circles) or presence (filled circles) of PME (30 μM). Currents were normalized to the currents maximally activated by 1 μM capsaicin (CAP) in the absence of PME. The figure shows averaged data fitted to the Hill equation. EC_50_ = 42.6 nM and EC_50 _= 148.2 nM in the absence or presence of PME, respectively. Calcium-free extracellular solutions were used to prevent TRPV1 desensitization in all experiments. Cells were perfused with capsaicin (20 nM) until the current reaching peak.

We also tested if another derivate of stilbenoid, PME, which shares a structure similar to resveratrol (Figure 1) could modulate TRP channels. In contrast to resveratrol, PME showed significant suppression on TRPV1 activity. In voltage-clamp experiments, low doses of capsaicin (20 nM) evoked small inward currents in the HEK293 cells. In the absence of extracellular calcium, no change was observed in the magnitude of the responses that were evoked by repetitive capsaicin applications (Figure 4A). After 2 min pretreatment with 30 μM PME, the same doses of capsaicin produced much smaller current responses (0.4 ± 0.1 fold, n=6 for PME; 0.9 ± 0.1 fold, n=8 for control without PME. *p* < 0.01) (Figures 4B and C). The suppression of *I*_CAP_ by PME was dose-dependent (Figure 4C). We confirmed that application of PME alone at 30 μM caused no change of membrane currents in HEK293 cells expressing rTRPV1. To examine how PME changes TRPV1 responsiveness, we measured *I*_CAP_ in single cells by serially applying a range of concentrations of capsaicin in the absence or presence of PME (30 μM). The currents were normalized to the maximal current (induced by capsaicin, 1 μM) without PME to each cell. Maximal currents in the presence of PME were almost the same as those obtained in the absence of PME. The resultant dose–response curves clearly demonstrate that PME suppresses TRPV1 responsiveness by increasing EC_50_ values without altering maximal responses (EC_50_ from 42.6 nM to 148.2 nM) (Figure 4D).

**Figure 5:**
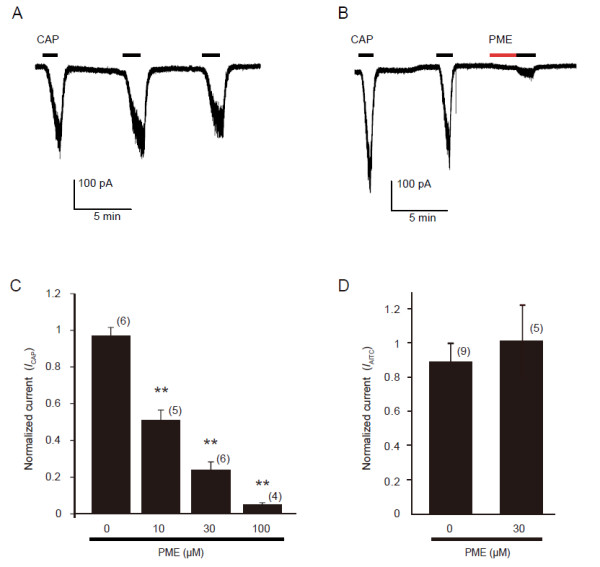
**PME inhibits *I***_**CAP **_**but not *I***_**AITC **_**in a concentration-dependent manner in DRG neurons. A**, **B**: Representative trace shows *I*_CAP _in the absence (**A**) or presence (**B**) of PME (30 μM). **C**: PME inhibited *I*_CAP _in a dose-dependent manner. ** p < 0.01 versus control, one-way ANOVA followed by Fisher’s PLSD. Cells were perfused with capsaicin (30 nM) until the current reaching peak in **A**-**C**. **D**: PME showed no inhibitory effect on *I*_AITC _(AITC, 100 μM). Numbers in parenthesis indicate cells tested. Calcium-free extracellular solutions were used to prevent TRPV1 desensitization in **A**-**C**.

We also recorded *I*_CAP_ on rat DRG neurons. Repeated treatment with capsaicin (30 nM) induced inward currents in small-sized cells with inconspicuous tachyphylaxis in the calcium-free extracellular solution (Figure 5A). We confirmed that application of PME alone at 30 μM caused no change of membrane currents in DRG neurons. In capsaicin-responsive neurons, much like in HEK293 cells that express TRPV1, we found that pre-treatment of PME (30 μM, 2 min) significantly inhibited *I*_CAP_ (Figure 5B). The suppression of the TRPV1 responsiveness by PME was dose-dependent (1.0 ± 0.1 fold, n=6 for control; 0.5 ± 0.1 fold, n=5 for PME at 10μM; 0.2 ± 0.1 fold, n=6 for PME 30 μM; 0.1 ± 0.01 fold, n=4 for PME 100μM; *p* < 0.01 vs. control for all concentrations) (Figure 5C). In contrast to resveratrol, PME (30 μM) did not show any effect on *I*_AITC_ in DRG neurons (0.9 ± 0.1 fold, n=9 for control, 1.0 ± 0.2, n=5 for PME, *p* > 0.05) (Figure 5D).

### Trans-stilbene showed no modulatory effect on either TRPA1 or TRPV1 channels

**Figure 6:**
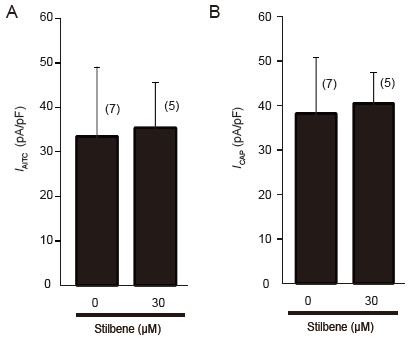
**Trans-stilbene shows no effect on either *I***_**AITC **_**or *I***_**CAP **_**in transfected HEK293 cells. A**: Bar graph shows the effect of trans-stilbene (30 μM, 3 min) on *I*_AITC _(AITC, 30 μM). (**B**) Bar graph shows the effect of trans-stilbene (30 μM, 3 min) on *I*_CAP _(capsaicin, 20 nM). Cells were perfused with either AITC or capsaicin until the currents reached their peak. Numbers in parentheses indicate cells tested.

Both resveratrol and PME are structural derivatives from stilbene (Figure 1). Resveratrol differs from trans-stilbene by having three hydroxyls attached to its phenyl groups, while PME differs from trans-stilbene by having a hydroxyl and a methoxyl attached to its phenyl groups. Both of the derivatives show inhibitory modulation on TRP channels. We then tested the effect of trans-stilbene with the basal stilbene structure on the TRP channels. Trans-stilbene was used at 30 μM, the same concentration as resveratrol and PME. In electrophysiological experiments, we found that trans-stilbene has no significant effect on either TRPA1 or TRPV1 in heterologous HEK293 cells. Pre-treatment with trans-stilbene for 3 min did not suppress either the *I*_AITC_ (AITC 30 μM) or *I*_CAP_ (capsaicin 20 nM) (*I*_AITC_ = −33.4 ± 13.3 pA/pF, n=7 for control; -35.3 ± 10.1 pA/pF, n=5 for trans-stilbene, p > 0.05) (*I*_CAP_ = −38.2 ± 12.5 pA/pF, n=7 for control; -40.4 ± 7.0 pA/pF, n=5 for trans-stilbene, p > 0.05) (Figure 6).

### Resveratrol/PME reduce the AITC-/capsaicin- induced nocifensive behaviors

TRPA1 and TRPV1 are known to be expressed on sensory neurons and act as an important component of pain. If resveratrol/PME can suppress the TRPA1/TRPV1 activation, pain sensation that is caused through the TRPA1/TRPV1 channels may also be suppressed by resveratrol/PME treatment. To this end, we performed intraplantar injections with AITC or capsaicin to the rat hind paw, and recorded the AITC- or capsaicin-induced nocifensive behaviors, respectively. Consistent with our previous studies, both AITC and capsaicin injections induced a significant flinch behavior of the injected hind paw during the 30 min post-injection period, whereas such behaviors were not observed in vehicle-injected rats (liquid paraffin or saline injection).

**Figure 7:**
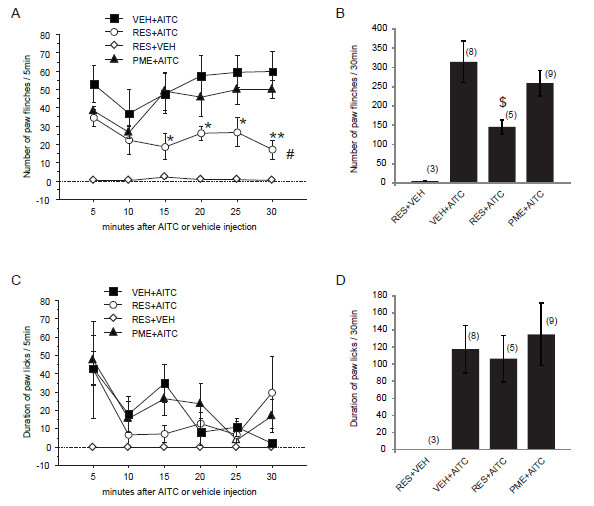
**Resveratrol but not PME inhibits AITC-induced pain behavior. **Resveratrol (RES, 300 μM, 50 μl) or PME (300 μM, 50 μl) was pre-treated subcutaneously 5 min before AITC (3% in 50 μl liquid paraffin) or vehicle (VEH) injection. **A**, **C**: Time course of the number of paw flinches (**A**) or duration of paw licks (**C**) induced by AITC or VEH after resveratrol or PME pretreatment, respectively. The number of flinches and duration of licks were counted per 5 min interval in the initial 30 min period after AITC injection. * p < 0.05, ** p < 0.01, versus VEH + AITC (one-way ANOVA followed by Fisher’s PLSD); # p < 0.05, versus VEH + AITC group (two-way repeated ANOVA followed by Fisher’s PLSD). **B**, **D**: Cumulative number of paw flinches (**B**) or duration of paw licks (**D**) in the initial 30 min period after AITC - or VEH - injection. $ p < 0.05, versus VEH + AITC, (one way ANOVA followed by Fisher’s PLSD). Numbers in parentheses indicate number of rats used in each group.

**Figure 8:**
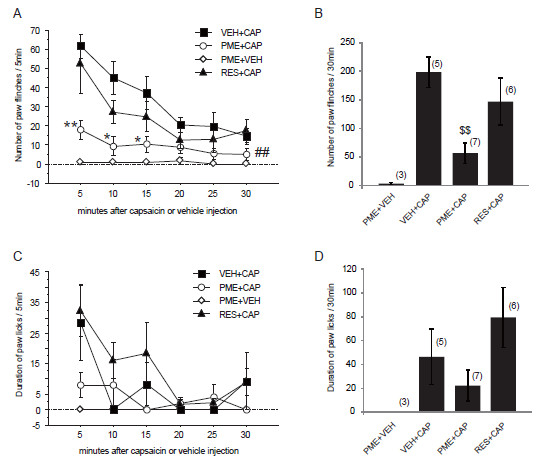
**PME but not resveratrol inhibits CAP-induced pain behavior. **PME (300 μM, 50 μl) or resveratrol (RES, 300 μM, 50 μl) was pre-treated subcutaneously 5 min before capsaicin (CAP, 0.05% in 50 μl saline with 0.5% tween20) or VEH injection. **A**, **C**: Time course of the number of paw flinches (**A**) or duration of paw licks (**C**) induced by capsaicin or VEH after PME or resveratrol pretreatment, respectively. The number of flinches and duration of licks were counted per 5 min interval in the initial 30 min period after capsaicin or VEH injection. * p < 0.05, ** p < 0.01, versus VEH + CAP (one-way ANOVA followed by Fisher’s PLSD); ## p < 0.01, versus VEH + CAP group (two-way repeated ANOVA followed by Fisher’s PLSD). **B**, **D**: Cumulative number of paw flinches (**B**) or duration of paw licks (**D**) in the initial 30 min period after capsaicin-injection. $$ p < 0.01, versus VEH + CAP (one way ANOVA followed by Fisher’s PLSD). Numbers in parentheses indicate number of rats used in each group.

Then we tested the effects of pretreatment resveratrol or PME on AITC- or capsaicin-induced behaviors. We found these two derivatives of stilbene did not cause any inflammatory reactions (e.g. redness, swelling) or acute nocifensive behavior (e.g. paw lifting, flinching or licking). After pre-treatment with resveratrol (300 μM) or PME (300 μM), AITC or capsaicin was injected into the same area of the hind paw, as previously described [30,31]. Pre-treatment with resveratrol significantly decreased the number of paw flinches induced by AITC (but not capsaicin) from 15 min post-injection period in a continuously gradual manner (Figure 7A, B). Pre-treatment with PME also significantly decreased the number of paw flinches induced by capsaicin (but not AITC) injection (Figure 8A, B). However, the AITC- or capsaicin-induced paw licks were not significantly changed by pretreatment of either resveratrol or PME, respectively (Figure 7C, D and Figure 8C, D).

## Discussion

Resveratrol has been shown to have chemo-preventive, cardio-protective, anti-aging, anti-oxidative and anti-nociceptive properties [32]. For the first time, we are able to report that resveratrol is a potent inhibitor of TRPA1 *in vitro* and *in vivo*. Given that TRPA1 plays important roles in pain transduction and oxidative stress response [28,33,34], our observations imply that the reported anti-nociceptive and anti-oxidative properties of resveratrol are possibly the results of the TRPA1 activity inhibition.

In this study, we found that resveratrol could inhibit the *I*_AITC_ in both heterologous HEK293 cells and DRG neurons in a dose-dependent manner. Two potential mechanisms are suggested to be involved in this inhibitory regulation. One would be that resveratrol inhibits the single channel conductance or open probability. The other would be that resveratrol decreases the channel density of TRPA1 on cell membranes. In the present study, the concentration-response curves of AITC resulting from the effect of resveratrol (Figure 2C) revealed that resveratrol suppressed the maximal response of AITC without conspicuously altering EC_50_, indicating that resveratrol inhibits TRPA1 activity through a non-competitive (to AITC) mechanism. Resveratrol might inhibit TRPA1 intracellularly by affecting the covalent modification of cysteine residues by AITC in the channel protein and subsequently inhibiting the channel conductance or open probability. However, the lack of effect of resveratrol on the EC_50_ for AITC-induced currents (Figure 2C), in addition to that resveratrol also suppressed the 2APB-induced currents (Figure 2D, E), indicated that the covalent modification of cysteine residues was not involved in the resveratrol-induced inhibition of TRPA1. Otherwise, resveratrol may decrease the channel density on cell membranes through promoting receptor internalization or by inhibiting membrane insertion of TRPA1 channels. TRPA1 may cycle between the plasma membrane and intracellular compartments, and the balance between membrane insertion and retrieval determines its surface abundance and activity [35]. A recent report indicated that TRPA1 channel desensitization in sensory neurons was regulated by internalization of itself [36]. On the other hand, PME inhibited *I*_CAP_. The concentration-response curves of capsaicin resulting from the effect of PME (Figure 4D) revealed that PME increased the EC_50_ of capsaicin without conspicuously altering maximal response, indicating that the PME inhibition of TRPV1 through a competitive (to capsaicin) mechanism, which differs from the resveratrol inhibition TRPA1. PME may supress the TRPV1 binding affinity for capsaicin, or modify the channel’s responsiveness to its own ligand. Anyway, the detailed mechanisms of resveratrol/PME regulation on TRPA1/TRPV1 requires further study.

In the present study, consistent with the patch-clamp data, topical treatment of resveratrol or PME produced a significantly persistent suppression of the number of paw flinches induced by intraplantar injection of AITC or capsaicin, respectively (Figure 7, 8), indicating resveratrol (or PME) can suppress the TRPA1 (or TRPV1) activity not only *in vitro* but also *in vivo*. Resveratrol has been reported to have analgesic properties against acute and chronic pain that is triggered either by nocifensive stimuli, inflammation or nerve injury [9-14]. As TRPA1 and TRPV1 in DRG neurons plays important roles in pain transmission and neurogenic inflammation, our results indicate that inhibition of *I*_AITC_ by resveratrol, as well as inhibition of *I*_CAP_ by PME, may at least partly account for its analgesic effects observed in these *in vivo* studies. These stilbenoids then might be considered as emerging targets for antinociceptive and anti-inflammatory pharmacotherapy.

Resveratrol has been shown to have anti-oxidative, anti-inflammatory, anti-proliferative, anti-angiogenic, and chemo-protective effects, with those on oxidative stress being the most important to human health, and possibly the foundation for some of the other beneficial effects. However, many signaling pathways are among the molecular targets of resveratrol, and thus we cannot be certain exactly what these pathways are at the present time. In concert, these effects may be beneficial in many disorders, particularly in diseases where oxidative stress plays an important role [37]. Oxidative stress results from metabolic activity or environmental stimuli, and produces highly reactive chemicals (e.g., H_2_O_2_) and oxidizing lipid products (e.g., 4-hydroxynonenal), which are known to activate TRPA1 and subsequently impacts human health [28,33,38]. Therefore, our finding that resveratrol inhibits TRPA1 activity may also provide a potential mechanism for the well-known anti-oxidative properties of resveratrol.

Resveratrol, as well as PME, was initially characterized as a phytoalexin, which is an anti-microbial substance synthesized by plants in response to infection [39]. Both of these derivatives of stilbene can inhibit TRP channels. It would be interesting to determine if the basal stilbene structure itself can modulate TRP activity. In the present study, trans-stilbene did not significantly inhibit either TRPA1 or TRPV1 (Figure 6), indicating that the modulation mechanism of these stilbenoids may not result from the direct binding of the basal structure of stilbene with the TRP domain. An indirect mechanism (for example, via the intracellular signals downstream stilbenoids) to explain the modulation of TRP channels by stilbenoids is suggested and needs to be further investigated.

## Conclusion

TRPA1 and TRPV1 are widely expressed channel present in many organs and tissues, including the respiratory system, cardiovascular system, gastrointestinal tract, inner ear, skin, skeletal muscle, dental pulp, pancreas, as well as the peripheral and central nervous system. These channels play important roles in inflammation, oxidative stress, and pain signals [40,41]. In the present study, we demonstrated for the first time that resveratrol and PME can significantly inhibit the TRPA1 and TRPV1 activity *in vitro* and *in vivo*, respectively. Therefore, the reported anti-nociceptive activities of resveratrol and PME may be considered to originate from TRP channel inhibition.

## Methods

### Animals and agents

All animal experimental procedures were approved by the Hyogo University of Health Sciences Committee on Animal Research (#2010-22) and were performed in accordance with the National Institutes of Health guidelines on animal care. Adult male Sprague–Dawley (SD) rats (100–250 g; Japan SLC, Inc, Shizuoka, Japan) were used for DRG culture and behavioral studies. Resveratrol (Sigma-Aldrich, St Louis, MO, USA), trans-stilbene (Sigma-Aldrich) and PME (Oy Arbonova Ab, Turku, Finland) were prepared from methanol based stock solutions that had been kept for not longer than 1 month at 4°C. Otherwise, capsaicin was prepared from ethanol based stock solution that was kept at −20°C. All compounds were added from these stock solutions to the recording solution. The final ethanol or methanol concentration in recording solutions was less than 0.1%. AITC was from Nacalai tesque (Kyoto, Japan). 2APB was from Sigma. Glutamax, fetal bovine serum (FBS), Penicillin–Streptomycin, Earle’s balanced salt solution (EBSS, Sigma), DMEM, vitamin solution and OPTI-MEM were from Invitrogen (Carlsbad, CA, USA).

### Mammalian cell culture

Human embryonic kidney-derived (HEK293) cells were maintained in DMEM (supplemented with 10% FBS, 2 mM glutamax, Penicillin and Streptomycin) and transfected with 1 μg of a mouse TRPA1 or a rat TRPV1 (mTRPA1 and rTRPV1 respectively) cDNA by using Lipofectamine LTX (Invitrogen). The cDNAs were generous gifts from Dr. Makoto Tominaga. To identify transfected cells, an enhanced green fluorescence protein reporter plasmid (BD Bioscience Clontech, CA) was also transfected at 0.1 μg. For primary culture of DRG neurons, whole DRGs were collected from the adult SD rats (100–150 g) using sterile techniques, and placed in ice-cold EBSS. Adhering fat and connective tissues were removed in order to isolate the DRGs. Each DRG neuron was then immediately placed in a medium consisting of 2ml of EBSS and 1.25 mg/ml of collagenase P (Sigma), and kept at 37°C for 60 min with occasional agitation. After dissociation of the DRG cells, this cell suspension was centrifuged for 5 min at 1000 rpm and the cell pellet was re-suspended in EBSS supplemented with 10% FBS, 2mM Glutamax, Penicillin, Streptomycin and vitamin solution. Recombinant rat nerve growth factor (100 ng/ml, Sigma) was added to the medium.

### Electrophysiology

Whole-cell patch-clamp recordings were performed 2 days after transfection of mTRPA1 cDNA, or 1 day after transfection of rTRPV1 cDNA to HEK293 cells, or 1 day after dissociation of the DRG neurons. Voltage-clamp experiments were performed at -60mV holding potential, and recordings were sampled at 5 kHz and filtered at 2 kHz. Current densities (pA/pF) and normalized currents were measured. The current magnitude was quantified by peak current amplitude in all experiments. A normalized current was obtained from dividing the third application-induced current to the second one, and adopted just in case the magnitude of the second current by agonist was larger than a half of the first current. In experiments with DRG neurons, after AITC was applied, capsaicin (1 μM) was applied at the end of the recording to identify whether *I*_AITC_ was mediated by TRPA1 channels. Data were obtained in the event that the DRG neurons were sensitive to both AITC and capsaicin applications, since an *I*_AITC_ in capsaicin-sensitive DRG neurons is certainly a TRPA1-mediated event [29]. The standard bath solution contained 140 mM NaCl, 5mM KCl, 2 mM MgCl_2_, 2 mM CaCl_2_, 10 mM HEPES and 10 mM glucose, pH 7.4 (adjusted with NaOH). In some experiment, the calcium-free bath solution contained 140 mM NaCl, 5 mM KCl, 2 mM MgCl_2_, 5 mM EGTA, 10 mM HEPES and 10 mM glucose, pH 7.4 (adjusted with NaOH) was used. The pipette solution contained 140 mM KCl, 5 mM MgATP, 5 mM EGTA and 10 mM HEPES, pH 7.2 (adjusted with KOH). The concentration-response curves for the effect of resveratrol on *I*_AITC_ densities (pA/pF) or PME on *I*_CAP_ densities were fit by the Hill equation using KaleidaGraph 4.1 (Synergy Software, PA, USA). All patch-clamp experiments were performed at room temperature (~25°C; RT). The solutions containing drugs were applied to the chamber (1 ml) by a gravity system at a flow rate of 4–5 ml/min.

### Behavioral studies

Twenty-five or twenty-one male adult SD rats (200–250 g) were used for the AITC- or capsaicin-induced nocifensive behavioral analyses, respectively. Behavioral studies were performed in accordance with the procedure of our previous reports [30,31]. To measure the effect of resveratrol (or PME) on AITC-induced nocifensive behavior, after adaptation, 50 μl resveratrol (300 μM, in saline) or PME (300 μM, in saline) was injected intraplantarly into the rat right hind paw. Five minutes after these injections, rats received intradermal injection of 50 μl of AITC (3% in liquid paraffin) to the same area of resveratrol injected plantar surface. To measure the effect of PME (or resveratrol) on capsaicin-induced nocifensive behavior, after adaptation, 50 μl PME (300 μM, in saline) or resveratrol (300 μM, in saline) was injected in the same manner as above. Five minutes after these injections, rats received intradermal injection of 50 μl of capsaicin (0.05% in saline with 0.5% Tween20). The rats were placed in a wire mesh cage immediately after the AITC or capsaicin injection, and the number of hind paw flinches and duration of licks per 5 min interval during the initial 30 min post-injection period was measured. The total number of flinches and total duration of licks during the entire initial 30 min for AITC or capsaicin was also calculated.

### Statistical analysis

All results are expressed as mean ± SEM. An unpaired *t*-test or one-way ANOVA was used to compare the electrophysiological data between the groups. One-way or two-way repeated ANOVA followed by Fisher’s PLSD was applied to the behavioral data. A difference was accepted as significant if the probability was less than 5% (p < 0.05).

## Competing interests

The authors declare that they have no competing interests.

## Authors’ contributions

LY carried out the electrophysiological and behavioral studies. SW performed the statistical analysis and participated in drafting the manuscript. YK participated in the behavioral studies. SY participated in the electrophysiological studies. KN supervised the project and edited the manuscript. YD conceived of the project, designed and coordinated the studies, and drafted the manuscript. All authors contributed to data interpretation, have read and approved the final manuscript.
